# Can we measure whether asthma guidelines lead to improved care?

**DOI:** 10.1038/s41533-024-00379-6

**Published:** 2024-06-27

**Authors:** Ronnie Tan, Anna Murphy, Chris Brightling, Dominick Shaw

**Affiliations:** 1 Department of Respiratory Sciences, University of Leicester, Institute for Lung Health, NIHR Respiratory Biomedical Research Centre, Leicester, UK; 2 Department of Respiratory Medicine, University Hospitals of Leicester NHS trust, Leicester, UK

**Keywords:** Asthma, Health policy

## Abstract

The British Thoracic Society (BTS) and Scottish Intercollege Guidelines Network (SIGN), as well as National Institute for Health and Care Excellence (NICE), have previously produced separate asthma guidance differing in some key aspects in diagnosis and management leading to confusion, potentially hampering guideline dissemination and uptake. While there are inherent challenges, the upcoming release of new joint BTS/SIGN/NICE asthma guidance presents an opportunity to assess guideline adoption and its impact on clinical practice. The use of prescription data via databases such as OpenPrescribing can be used as a surrogate for guideline adoption and potentially linked to clinical outcomes such as hospital episode statistics (HES). The potential recommendation for anti-inflammatory reliever therapy (AIR) and maintenance and reliever therapy (MART) with inhaled corticosteroid/formoterol combination therapy in the next iteration of UK asthma guidance will require the accurate coding for the respective therapeutic approaches on prescribing platforms in order to assess their impact in real-life clinical practice. This could then direct targeted measures to improve wider guidance adoption leading to better clinical care in asthma based on up to date evidence.

Clinicians, researchers and policy makers are anticipating the release of new joint British Thoracic Society/ Scottish Intercollege Guidelines Network/National Institute for Health and Care Excellence (BTS/SIGN/NICE) asthma guidelines. BTS and SIGN have produced combined guidance for many years, releasing their latest iteration in 2016, whilst NICE released new separate asthma guidelines in 2017. Unfortunately the guidelines differed in some key aspects leading to confusion, potentially hampering guideline dissemination and uptake^1,2^.

The initial justification for separate NICE guidance was rising drug costs, static death rates (although death rates were falling^3^) and issues with both over and under diagnosis, so a guideline approach that included consideration of health economics was felt justified. However, NICE guidance differed in multiple aspects to that of the BTS/SIGN, which led to confusion, and potentially poor guideline uptake^2^. For example, for asthma diagnosis, BTS/SIGN classified the probability of asthma into ‘high’, ‘intermediate’ or ‘low’ based on initial clinical assessment and physician judgement, whereas NICE emphasised the use of objective testing, including spirometry and more controversially, the measurement of exhaled nitric oxide (FeNO), with less consideration for issues of resource availability and capability within primary care. For initial add-on therapy, NICE recommended the addition of montelukast instead of a long-acting beta-2 agonist (LABA) to an inhaled corticosteroid (ICS), on the basis of cost reduction, notwithstanding the fact that montelukast is less effective than ICS/LABA combination therapy in reducing severe exacerbations and has a different safety profile. These differences in guidance led the Primary Care Respiratory Society to release its own consensus to support primary care doctors.

The impact of any guideline is likely down to the evidence presented in the guidelines themselves (although separate guidelines may cause confusion), but more importantly guideline uptake and their capacity to alter clinicians’ behaviour^1^. The release of the recent BTS “asthma attack bundle”, accompanied by a recorded webinar, is one attempt to positively influence clinicians’ behaviour. NICE previously recognised the potential limitations in adopting its guidance, stating “… primary care services should implement what they can of the recommendations, using currently available approaches to diagnosis until the infrastructure for objective testing in place”, reinforcing the need for adequate education, training and resources, for the effective adoption of clinical guidelines.

Establishing whether guideline advice leads to a change in clinical practice is not simple. NICE produces impact reports but these summarise clinical data rather than show a clear link between guidance and subsequent change in practice. The 2020 NICE impact statement on respiratory conditions^4^ describes a welcome increase in the number of patients with a written asthma action plan (based on data from the Asthma UK 2019 annual survey) and in the number of people who had their asthma control monitored at annual reviews (from the 2018/19 quality outcomes framework). One paper demonstrated an increase in the prevalence of COPD following NICE COPD guidelines based on analysis of the Health Improvement Network^5^. These are important improvements but whether they relate to the impact of NICE guidance is unclear and the evidence base for measuring guideline impact is limited. Other countries have data on guideline impact: The Japanese Asthma and Prevention Guidelines, regularly revised by the Japanese Society of Allergology, have contributed to the increased use of inhaled corticosteroids, which is thought to have reduced asthma mortality in Japan^6^. Finland, through its comprehensive National Asthma Programme, has also reduced the healthcare burden associated with asthma whilst reducing asthma-related healthcare costs concomitantly^7^. These interventions show that changing clinician behaviour based on the best available evidence is crucial in asthma, where safe effective treatment is available, and that establishing the correct diagnosis can prevent years of potentially expensive therapy being administered.

What other methods are available to gauge guideline impact? Measuring prescribing behaviour is now feasible via databases including OpenPrescribing^8^, which can be interrogated to establish prescribing patterns following guideline release. Given the difference between the BTS/SIGN and NICE asthma guidelines on the earlier use of montelukast in the treatment steps, prior to the use of ICS/LABA combination therapy^9^, we used OpenPrescribing (https://openprescribing.net/) to examine trends in prescription of montelukast as a surrogate for guideline adoption. We could not find any noticeable difference in montelukast use around the time of NICE guideline dissemination although the analysis was complicated by the changing denominator over time from items prescribed per 1000 population (2012–2018) to items prescribed per 1000 list size (2019–2023) so the data were not directly comparable. However, the slope of the graph was unchanged, with no obvious inflection point in 2017 when the NICE guidance was released, suggesting no real difference in prescribing behaviour—see Figs. 1 and 2. This gradual increase in use may instead reflect that montelukast (trade name Singulair) came off patent in 2012. Our analyses of other data sources including NHS Business Services Authority (NHSBSA) ePACT 2 using sub-ICB location (SICBL) in national average values, show a similar trend with no clear demarcation pre- and post-NICE guideline introduction.

**Fig. 1:**
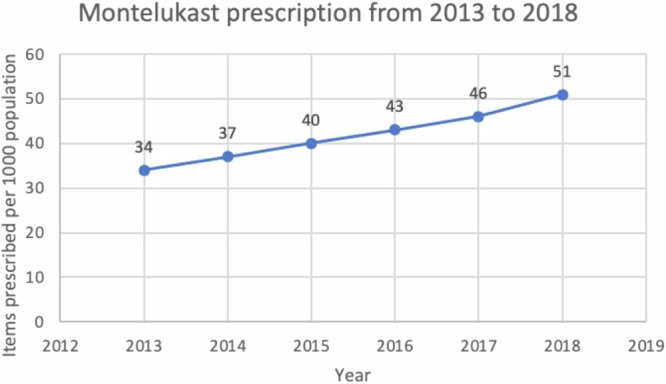
Prescription of montelukast per 1000 population size from 2013 to 2018. Data extracted from OpenPrescribing.net, Bennett Institute for Applied Data Science, University of Oxford, 2024.

**Fig. 2:**
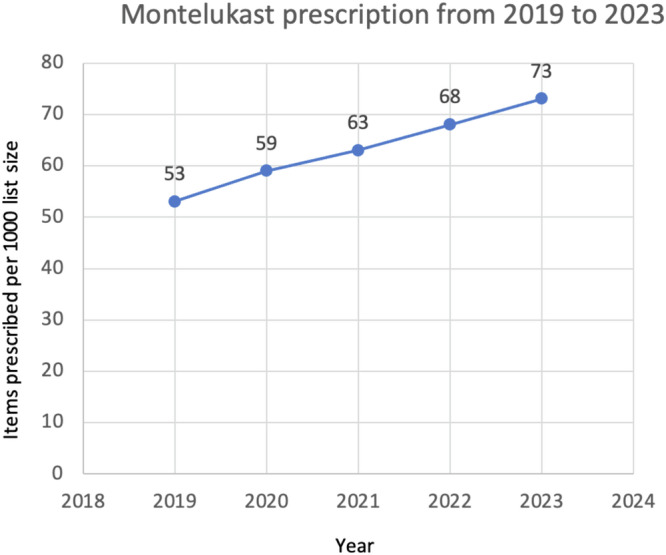
Prescription of montelukast per 1000 list size from 2019 to 2023. Data extracted from OpenPrescribing.net, Bennett Institute for Applied Data Science, University of Oxford, 2024.

Understanding guidance impact is especially important if a substantial change in established practice is recommended. It is possible that the next iteration of UK asthma guidance may follow the latest international Global Initiative for Asthma (GINA) guidance and recommend anti-inflammatory reliever therapy (AIR), as well as maintenance and reliever therapy (MART), which are both based on ICS/formoterol combination therapy. AIR in particular would represent a major step change in asthma management in the UK. AIR is already recommended in the recent short guidelines from the European Respiratory Society, which endorse that adults with mild asthma use as-needed ICS/formoterol, instead of regular ICS maintenance treatment plus as-needed short-acting beta-2-agonists (SABAs), and that adolescents with mild asthma use either as-needed ICS/formoterol or ICS maintenance treatment plus as-needed SABA^10^ .

The use of ICS/formoterol as AIR is also endorsed by other respiratory societies, because of a reduction in asthma exacerbations compared to traditional treatment algorithms seen in randomised controlled trials, and possibly reduces SABA over use^11–13^. These potential benefits need to be balanced by advice from the ERS statement^10^, that in adolescents with low or worsening lung function, regular ICS treatment rather than AIR should be considered. This lung function decline may be linked to FeNO level, which were higher in the as-needed ICS/formoterol groups, a link seen in other studies^14,15^. The use of AIR is also based on the assumption that a diagnosis of asthma is correct which is difficult to prove especially in primary care populations^16^.

The UK is not the only country to grapple with the impact of asthma guidelines. New Zealand, where much of the work on AIR and MART was developed, has recently assessed its use of ICS/LABA combinations following guideline changes. The results were encouraging with a temporal increase in ICS/LABA use^17^. However, there are important caveats. Firstly, albuterol was still the most prescribed inhaler and secondly a clear link to the GINA guideline update could not be made^18^.

If BTS/SIGN/NICE recommend a move to AIR, how could the impact of this step change be measured nationally? Prescribing data would be the simplest to track, although this is complicated by the current lack of a prescribing code for AIR. Read codes for MART exist already: situation (SCTID: 919601000000107) and procedure (SCTID: 922341000000101), but these codes also require them to be added to the prescription either by the prescriber or incorporated into the prescribing template. Importantly, careful delineation between the two prescribing approaches is needed to avoid confusion. Disambiguation of salbutamol prescribing for asthma and COPD will also be imperative to allow accurate analysis of its prescription trend in relation to AIR use in asthma alone. OpenPrescribing could then be used to track salbutamol use and ICS/formoterol combination prescriptions with a pre and post guideline analysis, and more widely Clinical Practice Research Datalink (CPRD) and hospital episode statistics (HES) could be interrogated to track asthma diagnoses, exacerbations and admissions. At a local level, prescribing audits, benchmarking and formulary changes can be collated. Assessing whether or not guidance leads to change in practice could direct the need for incentivisation of AIR/MART therapy in primary care (e.g. through Quality Outcomes Framework [QOF] metrics and/or locally agreed ICB medicine optimisation incentives guided by rational and robust clinical data and evidence) and help identify potential barriers to guideline implementation at a local level^19,20^.

We look forward to the new BTS/SIGN/NICE asthma guidance but suggest the following occur in tandem to ensure the impact of guidelines can be assessed and interventions targeted appropriately. (1) An ability to code separately on prescribing platforms when prescribing for AIR, MART and regular maintenance ICS/LABA and subsequently have separate analysis data on OpenPrescribing/CPRD etc., (2) a pre-planned assessment of BTS/SIGN/NICE guideline adoption, specified within the guidance to ensure uptake can be measured and potentially linked to clinical outcomes such as HES (perhaps performed by the British Thoracic Society) and (3) suggested measures to target geographical areas of poor guideline uptake. Together these measures should lead to further improvements in asthma care and ensure that all our patients benefit from the most up to date evidence.

## Data Availability

The dataset analysed during the current study is available at OpenPrescribing.net, Bennett Institute for Applied Data Science, University of Oxford, 2024.

## References

[1] Fowler SJ, O’Byrne PM, Buhl R, Shaw D (2018). Two pathways, one patient; UK asthma guidelines. Thorax.

[2] Keeley D, Baxter N (2018). Conflicting asthma guidelines cause confusion in primary care. BMJ.

[3] Shaw DE, Gaynor CM, Fogarty AW (2019). Changes in asthma mortality in England and Wales since 2001. Thorax.

[4] NICE. NICE impact respiratory conditions, Reviewing the impact of our guidance, Measuring the use of NICE guidance, Into practice, What we do, About, NICE. https://www.nice.org.uk/about/what-we-do/into-practice/measuring-the-use-of-nice-guidance/impact-of-our-guidance/nice-impact-respiratory-conditions (2024).

[5] Smith CJP, Gribbin J, Challen KB, Hubbard RB (2008). The impact of the 2004 NICE guideline and 2003 General Medical Services contract on COPD in primary care in the UK. QJM.

[6] Niimi A (2023). Executive summary: Japanese guidelines for adult asthma (JGL) 2021. Allergol. Int..

[7] Haahtela T (2006). A 10 year asthma programme in Finland: major change for the better. Thorax.

[8] Walker AJ, Curtis HJ, Croker R, Bacon S, Goldacre B (2019). Measuring the impact of an open Web-based prescribing data analysis service on clinical practice: cohort study on NHS England data. J. Med. Internet Res..

[9] White J, Paton JY, Niven R, Pinnock H (2018). Guidelines for the diagnosis and management of asthma: a look at the key differences between BTS/SIGN and NICE. Thorax.

[10] Papi A (2023). European Respiratory Society short guidelines for the use of as-needed ICS/formoterol in mild asthma. Eur. Respir. J..

[11] Bateman ED (2018). As-needed budesonide-formoterol versus maintenance budesonide in mild asthma. N. Engl. J. Med..

[12] O’Byrne PM (2018). Inhaled combined budesonide-formoterol as needed in mild asthma. N. Engl. J. Med..

[13] Hatter, L. et al. Asthma control with ICS-formoterol reliever versus maintenance ICS and SABA reliever therapy: a post hoc analysis of two randomised controlled trials. *BMJ Open Respir. Res.* **9**, 10.1136/bmjresp-2022-001271 (2022). 10.1136/bmjresp-2022-001271 PMC9422833 36007980

[14] van Veen IH (2008). Exhaled nitric oxide predicts lung function decline in difficult-to-treat asthma. Eur. Respir. J..

[15] Jackson, D. J. et al. Reduction of daily maintenance inhaled corticosteroids in patients with severe eosinophilic asthma treated with benralizumab (SHAMAL): a randomised, multicentre, open-label, phase 4 study. *Lancet.*10.1016/S0140-6736(23)02284-5 (2023). 10.1016/S0140-6736(23)02284-5 38071986

[16] Aaron SD (2017). Reevaluation of diagnosis in adults with physician-diagnosed asthma. JAMA.

[17] Hatter L (2023). Patterns of asthma medication use in New Zealand after publication of National Asthma Guidelines. J. Allergy Clin. Immunol. Pract..

[18] Zaeh SE, Krings JG (2023). Tracking the trends in the adoption of reliever-only ICS-formoterol therapy and SMART. J. Allergy Clin. Immunol. Pract..

[19] Ahmed K (2021). What drives general practitioners in the UK to improve the quality of care? A systematic literature review. BMJ Open Qual..

[20] Khedmati ME (2022). Evaluating the effectiveness of a local primary care incentive scheme: a difference-in-differences study. Med. Care Res. Rev..

